# Supplementary material for: Phylogenetic Analysis of Bovine Respiratory Syncytial Virus (BRSV) Subgroups in Wallonia Region of Belgium in Relation to Current Vaccination Strategies
Source: Vaccines (Basel). 2025 Mar 11;13(3):298. doi: 10.3390/vaccines13030298 (PMC11946434; doi:10.3390/vaccines13030298)
Supplement: Supplementary file 1 [file vaccines-13-00298-s001.zip › vaccines-3481581-supplementary.pdf]

Supplementary Table S1. Complementary information of samples analyzed for the study of bovine respiratory syncytial virus in Wallonia (Belgium)

Subgroup	Isolate name	Sample Origin	Sex	Breed	Herd location (Post code)
II	B15.0798	FMV	NK	NK	NK
II	B16.1135	FMV	M	BBB	4560
II	B19.2066	FMV	F	Holstein	4950
II	B19.0641	FMV	F	BBB	6767
II	B19.0625	FMV	M	BBB	6680
II	B19.217872	ARSIA	M	BBB	NK
II	B19.223592	ARSIA	M	BBB	6642
II	B20.0929	FMV	F	Limousin	4910
II	B20.1010	FMV	M	BBB	6800
II	B20.1034	FMV	M	BBB	6680
II	B20.008301	ARSIA	M	BBB	6800
II	B20.016058	ARSIA	M	BBB	6640
II	B20.011100	ARSIA	M	BBB	NK
II	B20.014340	ARSIA	M	Maine Anjou	NK
II	B20.049310	ARSIA	F	BBB	5530
II	B20.083322	ARSIA	M	BBB	6830
II	B20.099113	ARSIA	M	BBB	6800
II	B20.099838	ARSIA	M	BBB	1325
II	B23.0738	FMV	M	BBB	7740
II	B23.0806	FMV	F	BBB	5310
II	MLV-A	Vaccine bottle			
II	MLV-B	Vaccine bottle			
III	B19.207920	ARSIA	M	BBB	NK
III	MLV-C	Vaccine bottle			
III	MLV-D	Vaccine bottle			
VIII	B16.1160	FMV	F	Holstein	4850
VIII	B16.1586	FMV	F	Normand	4130
VIII	B16.1730	FMV	M	Blonde d'Aquitaine	6800
VIII	B16.1764	FMV	M	BBB	6800
VIII	B16.1929	FMV	F	BBB	6840
VIII	B16.8588	FMV	M	BBB	6850
VIII	B16.2015	FMV	M	BBB	4452
VIII	B16.0768	FMV	M	BBB	6640
VIII	B17.1500	FMV	M	BBB	6640
VIII	B17.1700	FMV	M	BBB	4877
VIII	B17.1120	FMV	F	BBB	6800
VIII	B18.1876	FMV	M	BBB	6800
VIII	B18.2356	FMV	M	BBB	6987
VIII	B19.1372	FMV	F	Holstein	4606
VIII	B19.1391	FMV	M	Blonde d'Aquitaine	NK
VIII	B20.0943	FMV	F	BBB	4920
VIII	B20.071281	ARSIA	F	BBB	5680
VIII	B20.079380	ARSIA	F	BBB	5377
VIII	B20.082416	ARSIA	F	BBB	6971
VIII	B20.083718	ARSIA	F	BBB	5300
VIII	B20.086101	ARSIA	M	BBB	6950
VIII	B20.089547	ARSIA	F	BBB	4280
VIII	B21.0944	FMV	F	BBB	4920
VIII	B21.1110	FMV	F	BBB	6941
VIII	B21.0011	FMV	F	BBB	6640
VIII	B21.0379	FMV	F	BBB	4217
VIII	B22.0873	FMV	M	Holstein	4821
VIII	B22.0935	FMV	F	Limousin	NK
VIII	B22.1677	FMV	M	BBB	6800
VIII	B22.0119	FMV	M	BBB	4280

FMV = Faculty of veterinary medicine, ARSIA = Regional Association for Animal Identification and Health, NK = Not known, F = Female, M = Male, BBB = Belgian blue breed
